# From wave to wave: a Dutch national study on the long-term impact of COVID-19 on well-being and family visitation in nursing homes

**DOI:** 10.1186/s12877-021-02530-1

**Published:** 2021-10-22

**Authors:** Ramona Backhaus, Hilde Verbeek, Bram de Boer, Judith H. J. Urlings, Debby L. Gerritsen, Raymond T. C. M. Koopmans, Jan P. H. Hamers

**Affiliations:** 1 Department of Health Services Research, Care and Public Health Research Institute, Maastricht University, Maastricht, Netherlands; 2 Living Lab in Ageing and Long-Term Care, Maastricht, Netherlands; 3 Department of Primary and Community Care, Radboud University Nijmegen, Medical Center, Nijmegen, Netherlands; 4 Department of Primary and Community Care, Radboud University Nijmegen, Medical Center, Nijmegen & De Waalboog “Joachim en Anna,” Center for Specialized Geriatric Care, Nijmegen, Netherlands

**Keywords:** COVID-19, Nursing homes, Visitors, Policy, Visiting ban, Well-being

## Abstract

**Background:**

To protect nursing home residents, many governments around the world implemented blanket visitor bans in March and April 2020. As a consequence, family caregivers, friends, and volunteers were not allowed to enter nursing homes, while residents were not allowed to go out. Up until now, little is known on the long-term consequences and effects of visiting bans and re-opening of nursing homes. The aim of the study was to assess the long-term effects of the pandemic on residents, family members, and staff, and their preparedness for the next coronavirus wave.

**Methods:**

A mixed-methods approach was used, consisting of a questionnaire and analyses of documentation (local visiting protocols). Of the 76 nursing home locations that participated in a Dutch national pilot on welcoming visitors back into nursing homes, 64 participated in this follow-up study. Data were collected in September/October 2020. For each nursing home, one contact person completed the questionnaire. Descriptive statistics were calculated for quantitative questionnaire data. Data on open-ended questions, as well as data from the documentation, were analyzed thematically.

**Results:**

The study demonstrated that the consequences of strict visiting bans do not disappear at the moment the visiting ban is lifted. Although in October 2020, daily life in nursing homes was more “back to normal,” more than one-third of the respondents indicated that they still applied restrictions. Compared to the situation before the pandemic, fewer volunteers were working in the nursing homes, grandchildren visited their relative less often, and visits differed.

**Conclusions:**

Five months after the visiting ban in Dutch nursing homes had been lifted, it still had an impact on residents, family members, and staff. It is questionable whether nursing homes feel prepared for welcoming visitors in the case of new COVID-19 infections. Nursing homes indicated that they felt prepared for the next wave, while at the same time, they were particularly concerned about staff well-being and vitality. It seems wise to invest in staff well-being. In addition, it seems desirable to think about how to support nursing homes in seeking a balance between infection prevention and well-being of residents, family members, and staff.

## Background

Worldwide, nursing homes have experienced a disproportionately high share of all COVID-19 cases and deaths [1, 2]. At the start of the pandemic, early in 2020, little was known about COVID-19 and how to prevent infections and spread [3, 4]. To protect nursing home residents, many governments around the world implemented blanket visitors bans in March and April 2020. As a consequence, family caregivers, friends, and volunteers were not allowed to enter nursing homes, while residents were not allowed to go out [3, 5]. In addition, (health) professionals were often not allowed to enter the homes either (e.g., physiotherapists, psychologists, hairdressers) [5].

Early in the pandemic, anecdotal reports from practice indicated that the visiting ban might have serious, negative effects on residents [5]. During the year 2020, several studies were conducted that assessed the experiences of residents, family members, and staff in nursing homes. It became clear that the pandemic and its related visiting bans in nursing homes have resulted in severe disruptions of residents’ routines and led to feelings of anxiety, depression, and loneliness of residents [6, 7]. Feelings of guilt, fear, worry, and isolation also increased for family members [3, 8]. Nursing home staff has experienced high levels of stress, anxiety, and burden [6].

What started at the beginning of 2020 as an emergency situation, in which very strict measures to prevent COVID-19 infections and spread in nursing homes were taken, quickly appeared to be the “new normal.” In summer 2020, after the first corona virus wave, steps for returning to “normal” were undertaken in many countries. As nursing homes are the homes of people that were – in the absence of effective vaccinations – “most at-risk” for severe or deadly effects of the virus in the year 2020, returning to normal in nursing homes was seen as challenging [6]. While, right after the visiting bans, the re-opening of nursing homes was welcomed by all stakeholders [9], little is known on the long-term consequences and effects of visiting bans and re-opening of nursing homes.

The Netherlands was one of the first countries where, under very strict conditions set by the government, the visiting ban in nursing homes was lifted and the (long-term) impact was assessed scientifically in a national pilot [5]. The first findings of this pilot have already been published [5, 9]. In this follow-up study, the aim was to assess the long-term effects of the pandemic on residents, family members, and staff, and their preparedness for the next coronavirus wave.

## Methods

A mixed-methods approach was used, consisting of a questionnaire and analyses of documentation (i.e., local visiting protocols).

### Setting and sample

In total, the sample consists of 76 nursing home locations that participated in the Dutch national pilot [5], which can be divided into two groups: a group of 26 nursing homes that re-opened for visitors on May 11 [5], and a group of 50 nursing homes that were allowed to welcome back visitors as of May 25. Table 1 describes nursing home and resident characteristics. Baseline data on the characteristics of the 26 nursing homes that re-opened on May 11 (described in Table 1) were collected in May 2020 and were reported earlier [5]. Baseline-data on the characteristics of the 50 nursing homes that re-opened on May 25 were collected in June 2020. The 76 nursing homes were representative for the Netherlands, as they were randomly selected from all local health authority regions in the Netherlands.

**Table 1:** Characteristics of nursing homes and residents

Characteristics	Nursing homes that re-opened May 11 (*n* = 26; measured May 2020)	Nursing homes that re-opened May 25 (*n* = 47; measured June 2020)
Total number of beds in psychogeriatric wards (range):	1097 (0–136)^a^	1270 (0–136)
Total number of beds in somatic wards (range):	589 (0–54)^a^	1560 (0–277)
Total number of residents currently living in a psychogeriatric ward (range):	1049 (0–134)^a^	1193 (0–70)
Total number of residents currently living in a somatic ward (range):	584 (0–54)^a^	1535 (0–270)

^a^ The data were based on 25 nursing homes, as one nursing home with 76 beds and 72 residents did not distinguish between somatic/psychogeriatric wards and was therefore excluded from analyses

In each nursing home, one contact person was selected to fill out electronic questionnaires, providing information about the whole nursing home. The nursing homes selected a person (from their staff) they considered to be able to provide the most information about the visiting policy and the local protocol of the specific nursing home. Often, contact persons were nursing home managers or local quality or policy officers.

### Data collection

Data were collected in September/October 2020. Contact persons filled out electronic questionnaires. The electronic questionnaire covered questions (statements, open-ended questions) about a) the number of COVID-19 infections, b) visits, activities, and daily life in nursing homes, c) impact on well-being of residents, family members, and staff (compared to the situation during the lockdown), and d) dealing with COVID-19 infections. Contact persons were invited to share the local protocols.

### Analyses

Descriptive statistics were calculated for quantitative questionnaire data. Data on open-ended questions, as well as data from the documentation (i.e., local visiting protocols) were analyzed thematically within the research team.

### Ethical considerations

The study was conducted in accordance with Dutch law and the Declaration of Helsinki. The study protocol (2020–6549) was reviewed by the local Medical Ethics Review Committee “CMO Regio Arnhem Nijmegen,” which concluded that the study was not subject to the Medical Research Involving Human Subjects Act. Information about the study was provided via email to the participants. Participation was strictly voluntary. Participants gave informed consent to participate in the study and could withdraw from participation at any moment. A reminder was sent to non-responders.

## Results

In September/October 2020, 64 out of 76 (84%) nursing homes participated.

### COVID-19 infections

In October 2020, in 6 out of 64 nursing homes (9%) residents were infected (17 infections in total, 1–6 residents per nursing home). In 15 out of 64 nursing homes (23%), staff members were infected (34 infections in total, 1–6 staff members).

### Visits, activities, and daily life

Table 2 provides insight into visits, activities, and daily life in the nursing homes (*n* = 64) in October 2020. According to the respondents (e.g., nursing home managers), in two-thirds (66%) of the nursing homes, for most residents, visits looked like before the COVID-19 pandemic. In most homes, residents could do activities again and received, for example, the regular care from (para)medical staff. Respondents from 14 (22%) nursing homes indicated that their residents did not receive the regular assistance from volunteers.

**Table 2:** Visits, activities, and daily life in nursing homes (*n* = 64) in October

Nursing homes that agreed/disagreed with the following statements:	Yes (n, %)	No (n, %)
For most residents, visits look like before the pandemic (March 2020).	42 (66%)	22 (34%)
Most residents can do activities again.	59 (92%)	5 (8%)
Most residents can go outside if they want to (e.g., to walk outside).	63 (98%)	1 (2%)
Most resident can leave the nursing home ward to visit family.	60 (94%)	4 (6%)
Most residents receive regular care from (para)medics again.	63 (98%)	1 (2%)
Most residents can make use of a pedicure or hairdresser.	63 (98%)	1 (2%)
Most residents receive their regular assistance from volunteers again. (*n* = 63)	49 (78%)	14 (22%)

Analysis of local visiting protocols showed great variation between nursing homes, e.g., regarding the number of visitors (one designated visitor or several visitors), the frequency of visits per week, whether the visits were supervised by staff, and whether physical contact between residents and visitors was allowed.

### Impact on well-being of residents, family members and staff

Table 3 provides insight into the impact on well-being of residents, family members, and staff in October.

**Table 3:** Impact on well-being of residents, family members, and staff

Compared to the situation during the lockdown, residents now (September/October 2020). ..	More (n,%)	Same (n, %)	Less (n, %)	Unknown (n, %)
- have a more positive mood.	29 (45%)	27 (42%)	7 (11%)	1 (2%)
- eat and drink more.	7 (11%)	46 (72%)	3 (5%)	8 (13%)
- show less misunderstood behavior.	13 (20%)	36 (56%)	10 (16%)	5 (8%)
- are more active and seek more contact.	19 (30%)	39 (61%)	3 (5%)	3 (5%)
- have physical problems.	2 (3%)	53 (83%)	2 (3%)	7 (11%)
- show cognitive decline.	13 (20%)	35 (55%)	7 (11%)	9 (14%)
**Nursing homes that agreed/disagreed with the following statements about family members:**	**Yes (n, %)**		**No (n, %)**	
Most family members indicate that they are afraid of getting infected themselves.	12 (19%)		52 (81%)	
Most family members indicate that they are afraid of infecting others.	22 (34%)		42 (66%)	
Most family members keep 1.5 m distance from residents and others.	49 (77%)		15 (23%)	
Most family members do not visit the residents when they have symptoms of a common cold.	62 (97%)		2 (3%)	
Most family members take the hygiene measures into account.	61 (95%)		3 (5%)	
Since the re-opening for visitors, most family members visit their relative less frequently.	14 (22%)		50 (78%)	
**Nursing homes that agreed/disagreed with the following statements about staff members:**	**Yes (n, %)**		**No (n, %)**	
Most staff members indicate that they are afraid of getting infected themselves.	29 (45%)		35 (55%)	
Most staff members indicate that they are afraid of infecting others. (*n* = 59)	50 (85%)		9 (15%)	
Most staff members keep 1.5 m distance from each other and others.	49 (77%)		15 (23%)	
Most staff members do not work if they have symptoms of a common cold (without being tested for COVID-19).	58 (91%)		6 (9%)	
Most staff members take the hygiene measures into account.	64 (100%)		0 (0%)	
Most staff members feel safe during work.	58 (91%)		6 (9%)	
Most regular work meetings are taking place again.	42 (66%)		22 (34%)	
The extra work pressure due to the pandemic has decreased in the meantime.	21 (33%)		43 (67%)	

Respondents reported a better well-being of residents compared with the situation during the visiting ban. In 45% of the nursing homes, residents had a more positive mood compared to the situation during the visiting ban (lockdown). Thirty percent of the nursing homes said that residents now were more active and more actively sought contact with others. For some resident outcomes, mixed experiences were reported (e.g., residents showing less or more cognitive decline; see Table 4). While most respondents (81%) indicated that family members were not afraid of getting infected themselves, about one-third (34%) of the respondents indicated that family members were afraid of infecting others in the nursing home. Twenty-three percent of the respondents stressed that most family members did not keep to the 1.5 m distance measure, while other hygiene measures like wearing face masks were followed by family members in most nursing homes. One out of five (22%) respondents said that residents received fewer visits since the re-opening of the nursing homes.

**Table 4:** Dealing with COVID-19 infections and prevention of visitor bans (*n* = 62)

Nursing homes that agreed/disagreed with the following statements:	Yes (n, %)	No (n, %)
In case of one or more infection(s) within a ward, all residents and their relatives can decide for themselves whether or not they want to welcome visitors.	16 (26%)	46 (74%)
In case of one or more infection(s) within a ward, we will close the whole ward for visitors.	27 (44%)	35 (56%)
In case of one or more infection(s) within a ward, we will close the whole nursing home for visitors.	4 (6%)	58 (94%)
In case of one or more infection(s) within a ward, protective measures will also be sharpened within other nursing home locations of our organization.	19 (31%)	43 (69%)
In case of one or more infection(s) within a ward, the daily life of other (non-infected) residents does not change (e.g., daily routines, activities).	33 (53%)	29 (47%)
A resident suffering from COVID-19 is allowed to welcome visitors.	32 (52%)	30 (48%)
A resident suffering from COVID-19 will move to a quarantine ward.	16 (26%)	46 (74%)
In case a resident suffering from COVID-19 welcomes visitors, the resident has to wear protective equipment.	32 (52%)	30 (48%)
When visiting a resident suffering from COVID-19, the visitor has to wear protective equipment.	61 (98%)	1 (2%)
Residents in a terminal end-of-life phase are allowed to welcome visitors at all times (also in situations in which the ward is closed for visitors).	61 (98%)	1 (2%)
Visitors are, at all times, allowed to bid farewell from a resident according to their own preferences (e.g., rituals, physical contact).	43 (69%)	19 (31%)

The reported staff well-being was less positive. In most nursing homes, staff was afraid of getting infected (45%) and/or infecting others (85%). Nevertheless, only 9% of the respondents indicated that staff did not feel safe during work. All nursing homes reported that staff members took the hygiene measures into account. In two-thirds of the nursing homes (67%), the extra work pressure due to the pandemic had not decreased, according to respondents. Respondents indicated that nursing homes had to deal with higher absenteeism rates and that they had difficulties with scheduling their shifts. Reported reasons for higher absenteeism rates were related to staff suffering from long-covid or dealing with emotional difficulties (e.g., the emotional impact in wards where many residents had died due to COVID-19 or having fewer possibilities for (social) recreational activities). In addition, staff members had to wait a long time to be tested for COVID-19 or receive the test results. In case of negative test results, this meant that these staff members stayed at home unnecessarily long, leading to a higher workload for others. One-third of the nursing homes (34%) indicated that regular work meetings were not yet taking place again.

### Welcoming visitors in the case of COVID-19 infections

In October, respondents were asked to report on how their nursing home dealt with COVID-19 infections and what was done to prevent a ban on visitors (Table 4).

Nursing homes differed in how they dealt with infections (Table 4): in 26% of the nursing homes, residents and their relatives decided for themselves whether they wanted to welcome visitors in case of infection(s), while in 44% of the nursing homes, the whole ward would close for visitors. Respondents of 94% of the nursing homes indicated that they would not close the whole nursing home for visitors if there was an infection. In half of the homes (52%), residents suffering from COVID-19 were allowed to welcome visitors. Fifty-three percent of the nursing homes indicated that in the case of one or more infection(s), daily routines and activities of other, non-infected residents would not change.

### Dealing with the second coronavirus wave

In October, most respondents indicated that they were prepared for the next coronavirus wave. Most nursing homes had local protocols they could follow in case of infections. Nearly all nursing homes were equipped with sufficient protective equipment for residents (95%), staff members (98%), and volunteers (97%). Some nursing homes indicated that they did not have sufficient protective equipment for visitors (19%). The COVID-19 test capacity was more scarce. While 86% of the homes had sufficient capacity to test residents, nursing homes indicated that they had difficulties with the test capacity for staff members (34%) and volunteers (59%). Often, volunteers could not be tested at all.

## Discussion

This is one of the first studies providing insight into the long-term impact of the pandemic on residents, family members, and staff. The study demonstrated that the consequences of strict visiting bans do not disappear at the moment the visiting ban is lifted. Although, in October 2020, 5 months after the Dutch visiting ban, daily life in nursing homes was more “back to normal” (e.g., residents could do activities again, received regular (para)medical care again), at the same time, more than one-third of the respondents indicated that they still applied restrictions. Compared to the situation before the pandemic, fewer volunteers were working in the nursing homes, grandchildren did visit their relative less often, and visits differed (e.g., stricter rules related to visits). “Normality” had not been reached.

Respondents indicated that residents and family members benefited from the visits and that most family members did take into account the restrictive measures related to visiting. Staff members still had to deal with high work pressure, often related to high absence rates of staff (due to illness, long-covid, quarantine), after months with a promising downward trend in the number of COVID-19 infections in summer 2020 and increased attempts to offer psychological support to staff on how to deal with the work-related consequences of COVID-19 [4].

We saw a great variety in how nursing homes dealt with COVID-19 infections. While the Dutch government recommended that no more national visiting bans in nursing homes should be implemented [9], nearly half of the respondents indicated that they would close whole nursing home wards for visitors in case of an infection. A minority of the nursing homes would close the whole nursing home for visitors if there was an infection. This demonstrates that nursing homes figure out for themselves how to deal with infections, seeking their own balance between safety and resident well-being. Especially when nursing homes just had re-opened their doors for visitors, many restrictive measures had to be taken into consideration. Nevertheless, they could make their own choices in seeking a balance between infection prevention and well-being. At the same time, they had to realize that these choices did not only affect the residents, but also had serious consequences for family members and staff [10]. While many choices made in nursing homes may have consequences for family members and staff, it seems that the visiting ban made these “extra dimension” [10] of consequences more visible. Residents and family members were not involved in the decision of implementing a visiting ban [11]. The long-term consequences of seeing visits of family members as potentially harmful for infection prevention and a potential threat to residents’ health might have had a great impact on the relationships between residents, family members, and staff. Another recent Dutch study described the term “visitor” as a “euphemistic term” [10] concerning the residents’ loved ones (often a partner or child), who not only visit the resident, but also play an essential caregiving role.

Reflecting on the impact of the restrictive measures applied, Dutch client representatives and managers of nursing homes affiliated with the Living Lab in Ageing and Long-term Care argued that probably one of the most important lesson’s learned is not to apply a visitors ban again. Knowing that the life expectancy of nursing home residents on average is very limited, the focus should be on wellbeing. This particularly goes for future decision-making regarding infection prevention in nursing homes, in which residents, their relatives and nursing home staff should be highly involved [12]. This need for joint decision making and clear communication regarding COVID-19 infection prevention is also emphasized in a recent study from the UK that includes data directly derived from family carers of people living with dementia [13].

Although most respondents indicated that they felt prepared for the second wave, the study showed that the test capacity for staff and volunteers was often still scarce. Already early in the pandemic (spring/summer 2020), nursing homes were at the “end of the line” [4] with regard to national allocation of COVID-19 tests and personal protective equipment. For personal protective equipment, the shortage appears to have resolved compared to the period May–June 2020 [9]. Respondents were most concerned about the impact of this second wave on staff. At the moment of data collection, it was unknown when residents and staff members of Dutch nursing homes could get vaccinated against COVID-19. On January 18, 2021, the first residents of Dutch nursing homes were vaccinated [9], which might have a great impact on well-being, family visitation, and workload of staff in nursing homes.

Some methodological limitations must be considered. As we collected data from a contact person being able to provide most information about the nursing homes’ visiting policy, this contact person may have less insight into the actual impact of the visiting policy on residents, family members, and staff. Information from contact persons was collected via questionnaires, while conducting interviews would have enabled us to interpret the findings in more detail. Moreover, the national COVID-19 measures that were in force for all Dutch citizens might have an influence on the results of our study, as some respondents indicated that they did not feel the freedom to make their own choices with regard to how to deal with COVID-19 infections.

## Conclusions

Five months after the visiting ban in Dutch nursing homes had been lifted, the ban still had consequences on (the relationships of) residents, family members, and staff and “normality” had not been reached. Although the Dutch government decided that visiting bans in nursing homes should be prevented, it is questionable whether nursing homes feel prepared for welcoming visitors in case of new COVID-19 infections. Nursing homes indicated that they felt prepared for the next wave, while at the same time, they were particularly concerned about staff well-being and vitality. Since it is impossible to forecast the future impact of the pandemic, it seems wise to invest in staff well-being.

As we saw a great variety in how nursing homes deal with COVID-19 infections, it seems desirable to think about how to support them in making choices to seek a balance between infection prevention and well-being of residents, family members, and staff. The national COVID-19 measures that were in force for all Dutch citizens (e.g., measures with regard to the number of visitors or social isolation in case of COVID-19 infections) had an impact on the choices made by nursing homes, and many nursing home organizations did take little room to make own choices.

## Data Availability

The datasets generated and/or analyzed during the current study are not publicly available to ensure the privacy of participating nursing homes, but are available from the corresponding author on reasonable request.
